# Carryover effects of larval environment on individual variation in a facultatively diadromous fish

**DOI:** 10.1002/ece3.5582

**Published:** 2019-08-20

**Authors:** Grégoire Saboret, Travis Ingram

**Affiliations:** ^1^ Département de Biologie, Master Biosciences ENS de Lyon Lyon France; ^2^ Department of Zoology University of Otago Dunedin New Zealand

**Keywords:** Amphidromy, behavioral syndrome, common bully, *Gobiomorphus cotidianus*, individual specialization, life history, otolith microchemistry, partial migration

## Abstract

1. Intraspecific trait variation may result from “carryover effects” of variability of environments experienced at an earlier life stage. This phenomenon is particularly relevant in partially migrating populations composed of individuals with divergent early life histories. While many studies have addressed the causes of partial migration, few have investigated the consequences for between‐individual variability later in life.
2. We studied carryover effects of larval environment in a facultatively diadromous New Zealand fish, *Gobiomorphus cotidianus*, along an estuarine salinity gradient. We investigated the implications of varying environmental conditions during this critical stage of ontogeny for adult phenotype.
3. We inferred past environmental history of wild‐caught adult fish using otolith microchemistry (Sr/Ca) as a proxy for salinity. We tested for main and interactive effects of larval and adult environment on a suite of traits, including growth rates, behavior (exploration and activity), parasite load, and diet (stable isotopes and gut contents).
4. We found a Sr/Ca consistent with a continuum from freshwater to brackish environments, and with different trajectories from juvenile to adult habitat. Fish with Sr/Ca indicating upstream migration were more vulnerable to trematode infection, suggesting a mismatch to freshwater habitat. Diet analysis suggested an interactive effect of larval and adult environments on trophic position and diet preference, while behavioral traits were unrelated to environment at any life stage. Growth rates did not seem to be affected by past environment.
5. Overall, we show that early life environment can have multiple effects on adult performance and ecology, with the potential for lifetime fitness trade‐offs associated with life history. Our study highlights that even relatively minor variation in rearing conditions may be enough to generate individual variation in natural populations.

## Graphical abstract

We investigated carryover effects of larval environment in a facultatively diadromous New Zealand fish, *Gobiomorphus cotidianus*. Using otolith microchemistry (Sr/Ca) as a proxy for salinity, we found that larval environment influence adult diet (stable isotopes and gut content) and parasitism, with a near significant effect on juvenile growth and no effect on adult behavior (exploration and activity).

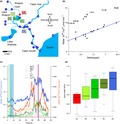

## 1 INTRODUCTION

Every event and experience during the lifetime of an individual has the potential to affect its traits later in life (Pechenik, 2006). It is increasingly recognized that individuals within a population could differ simply because they have encountered different environments during earlier life stages, a phenomenon referred to as “carryover effects” (Van Allen & Rudolf, 2016). Defined broadly, carryover effects occur when an experience impacts an individual's phenotype at a later time (O'Connor, Norris, Crossin, & Cooke, 2014), which can have implications for its performance and fitness. Early life environment therefore represents a possible mechanism generating among‐individual variation later in life, including individual niche variation and behavioral syndromes (Dall, Bell, Bolnick, & Ratnieks, 2012).

Carryover effects often occur when individuals can move between habitats throughout their lifetime. On the one hand, individuals can be affected by a “mismatch effect,” if this dispersal causes them to be maladapted to a new environment. For instance, snails that develop shell shapes that defend against specific predators are more vulnerable when later exposed to new predators (Hoverman & Relyea, 2009). On the other hand, individuals that benefit from a high‐quality environment early in their lives can experience higher fitness later in life in any habitat, a phenomenon known as the “silver spoon effect” (Van De Pol, Bruinzeel, Heg, Van Der Jeugd, & Verhulst, 2006). Carryover effects have been reported in many taxa, including ascidians (Van Allen, Briggs, McCoy, & Vonesh, 2010), insects (Arambourou, Sanmartín‐Villar, & Stoks, 2017), birds (Tarwater & Beissinger, 2012), mammals including humans (Pettorelli et al., 2002), and fish (Gallagher, Piccoli, & Secor, 2018; Gillanders, Izzo, Doubleday, & Ye, 2015; Shima & Swearer, 2010). These observations have been corroborated by controlled experiments; for example, manipulation of environment quality of tadpoles affects their ultimate fitness as adult frogs (Goater, 1994). Most studies have focused on carryover effects on fitness, while few if any have investigated between‐individual variation in other traits such as behavior, resource use, and trophic interactions (Bolnick et al., 2003; Dall et al., 2012).

The potential for carryover effects may be particularly relevant in species with a high degree of life‐history plasticity and the potential to rear in distinct environments. Partial amphidromy in fishes present an extreme case of flexible life history (Chapman et al., 2012). Amphidromous fishes hatch in freshwater, immediately migrate to the ocean as larvae, then return as juveniles to complete the majority of their growth in freshwater (McDowall, 2007). Some species are facultatively amphidromous, meaning that they can use a lake or estuary as an alternative pelagic rearing environment (Hicks et al., 2017). Partially amphidromous populations may therefore be composed of a mix of fish that reared in the ocean and others that remained in freshwater or brackish environments. While the causes of partial migration have been the focus of a large amount of research (Brodersen, Ådahl, Brönmark, & Hansson, 2008; Chapman, Brönmark, Nilsson, & Hansson, 2011; Kaitala, Kaitala, & Lundberg, 1993; Lundberg, 1988; Shaw & Levin, 2011), few studies have addressed carryover effects resulting from this flexible migratory strategy that can lead to co‐occurring adults that experienced very different environments early in life. For instance, studies of white perch have shown that migration of juveniles to the sea can deeply impact adult fitness, even years after migration (Gallagher et al., 2018). A full understanding of the implications of partial migration and flexible life history may therefore require consideration of carryover effects.

New Zealand's freshwater fish fauna is dominated by ancestrally amphidromous galaxiids and eleotrids. While some of these species have become permanently restricted to freshwater habitats and others remain obligately amphidromous, a few species including the common bully (*Gobiomorphus cotidianus*, McDowall 1975) are facultatively amphidromous and can rear in marine, estuarine or freshwater (McDowall, 1990a). Some populations are fully landlocked above dams or other barriers, but in coastal lakes open to the ocean bullies exhibit flexibility in larval rearing environment (Closs, Smith, Barry, & Markwitz, 2003; Hicks et al., 2017). The gradient from freshwater to brackish and saltwater represents a significant shift in environmental conditions. Salinity has numerous implications for ion balance, biomineralization, larval food (zooplankton) abundance and composition (David et al., 2016), and exposure to different predators and parasites (Blasco‐Costa, Koehler, Martin, & Poulin, 2013). Larvae migrating to higher salinity environments may benefit from greater productivity and resource availability (Gross, Coleman, & McDowall, 1988) and potentially reduced intraspecific competition (Lundberg, 1988), but may also experience mismatch effects due to the need to return to freshwater later in life. Lowland lakes such as Lake Waipori, in the Waipori–Taieri river system in Otago, New Zealand, therefore present a valuable opportunity to evaluate carryover effects due to variation in larval salinity environment.

We investigated the effects of early life experience on adult traits in common bullies in Lake Waipori. We hypothesized that variation in larval environment along the salinity gradient could carry over to produce variation in bully growth, behavior, and ecology. Specifically, we predicted that growth rate would be enhanced by higher salinity in early life due to a “silver spoon effect” (Gallagher et al., 2018). We predicted that variation in food availability in different larval environments could lead to variation in adult diet measured using gut contents and stable isotopes. We predicted that adult parasite load would be reflective of long‐term habitat use, but also that mismatch effects could occur if individuals encountered parasites that they were not exposed to early in life. Finally, we predicted that behavioral traits such as boldness or exploration would be correlated with the degree to which individuals had moved between habitats early in life. We used otolith trace element analysis to infer past salinity environments of wild‐caught fish and tested whether larval environment and adult environment interactively predicted individual variation in adult phenotypes.

## 2 MATERIALS AND METHODS

### 2.1 Study site

**Figure 1:**
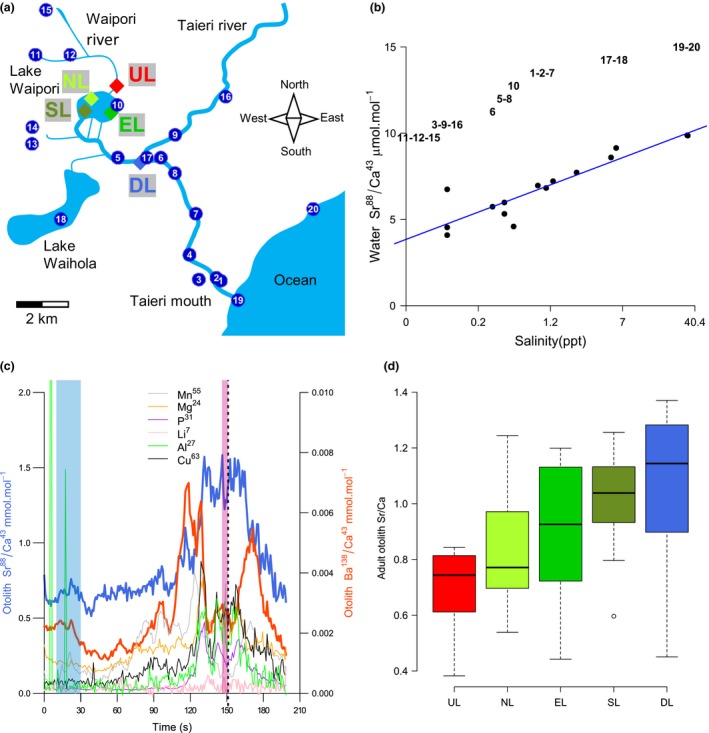
(a) Map of Lake Waipori and the lower Taieri and Waipori River systems. Blue circles: water samples; colored diamond: fish collection, referring to the five groups in (d) (b) Molar Sr/Ca in water samples compared to salinity. Numbers refer to sample locations in (a). Close locations (e.g., 6 and 17) can have very different water quality due to differences in sampling time, highlighting tidal influence downstream of Lake Waipori. (c) Trace element profiles in one‐year‐old fish otoliths of Sr/Ca (blue) and Ba/Ca (red). The core is characterized by a spike a Mn/Ca (gray) followed by a “clump” of P/Ca (purple) and Mg/Ca (orange). Larval (blue) and adult (purple) past Sr/Ca are represented by shaded areas. (d) Boxplot of adult otolith Sr/Ca of the five groups (a), from lower salinity to higher salinity

Lake Waipori is a part of the Waihola–Waipori wetland complex in the Taieri Plain, 30 km south of Dunedin, New Zealand. The 225 ha lake is shallow (1.2 m maximum depth) with a short average water residence time of 1.9 days (Schallenberg & Burns, 2003). The system has a complex hydrology, and the lake receives inputs from the Waipori River below the dammed Lake Mahinerangi, from floodwater pumped from nearby agricultural land, and from smaller streams and adjacent wetlands. As the lake is near sea level, it experiences tides despite being 10 km from the mouth of the Taieri River and is subject to occasional intrusions of brackish water as well as water from the Taieri River (Figure 1a). Fortnightly water quality measurements over a year (1997–1998) have previously shown a clear gradient of salinity along the main axis of Lake Waipori (Schallenberg & Burns, 2003), while sites upstream of the lake are largely pure freshwater. Salinity in the Waihola–Waipori wetland complex is associated with water quality variables such as pH, nutrient concentrations, and chlorophyll a, as well as zooplankton community composition (Schallenberg & Burns, 2003). We collected filtered water samples from various locations in the Taieri system on 15 January and 24 March 2018. Conductivity and salinity were measured in situ with a YSI Professional Plus multiprobe (YSI, Yellow Springs, OH, USA), and element concentrations were analyzed using inductively coupled plasma mass spectrometry at the Centre for Trace Element Analysis at the University of Otago.

### 2.2 Common bully collection

*Gobiomorphus cotidianus* is a small (30–120 mm total length [TL]), facultatively anadromous eleotrid fish that is endemic to New Zealand but is widely distributed throughout the country (McDowall, 1990b, Closs et al., 2003; Hicks et al., 2017; Michel et al., 2008). Larvae are pelagic while after metamorphosis (TL ~ 18–30 mm), fish occupy primarily benthic habitats in lakes and slow flowing streams (McDowall, 1990b). They prey on a wide range of invertebrates and are an important food source for native eels and introduced trout and perch. *Gobiomorphus cotidianus* is abundant in Lake Waipori and the larger Waihola–Waipori wetland complex and shows little to no population genetic structuring within this area (T. Ingram and T. King, unpublished data).

On 12–13 February 2018, a total of 158 adult common bullies were collected using Gee minnow traps set by boat at various locations in Lake Waipori (*N* = 131) as well as in the Waipori River above (*N* = 10) and below (*N* = 17) the lake (Figure 1a). These fish were collected in two subsets: one sampled to prioritize diet (gut content and stable isotope) analysis, and the other to allow behavioral trials and measurement of parasite load. The “diet” fish were collected in traps set for periods of 2–3 hr during the day, to ensure that gut contents reflected primarily prey consumed before entering the trap. These fish were euthanized with an overdose of AQUI‐S 20E (AQUI‐S New Zealand) and frozen at −20°C. Concurrently, samples of potential prey items were collected from various locations in the lake using a sweep net, then frozen. The “behavior” fish were caught in traps left overnight and transported the following day in coolers of lake water to the Department of Zoology at the University of Otago. All procedures involving animals were approved by the University of Otago Animal Ethics Committee (protocol 104/17).

### 2.3 Behavioral assays and parasitism

“Behavior” fish were housed in 20 L glass aquaria with 3–6 fish per aquarium and fed ad libitum with frozen chironomid larvae. The temperature in the room was set to 15°C, the photoperiod was set to 12:12 hr day/night with a gradual 2‐hr transition at “dawn” and “dusk,” and sections of PVC pipes were provided as refuges. Each fish was given one subcutaneous visual implant elastomer tag (Northwest Marine Systems) to distinguish it from other fish in it s tank, using a unique combination of one of two colors (yellow and orange) and one of three tag positions (left or right dorsal postcranial, or ventral).

After a one week acclimation period, fish were subjected to two standard noninvasive behavioral assays to measure activity and exploration (Dingemanse et al., 2007). Each fish was assayed first for activity then for behavior one or two days later, then both assays were repeated one week later. No feeding occurred on testing days until after trials were completed. Trials were carried out in opaque plastic boxes (356 × 257 × 185 mm length × width × depth), filled with water from the fish's home aquarium to a depth of 70 mm. Trials were filmed from above with GoPro Hero 5 cameras (GoPro Inc.) at 60 fps, using a linear field of view to minimize distortion and controlled by remote to minimize disturbance to the fish. Video analysis was done using EthoVision XT (version 11.5; Noldus Information Technology), with tracking success at or near 100% for all trials. For the activity assay, fish were placed in an empty arena and video recorded for 10 min, with the mean velocity (m/s) calculated in EthoVision as the total distance travelled divided by the total duration of the trial. For the exploration trial, two white plastic corflute sheets were placed 84 mm from one end of the arena, with the fish initially introduced to this confined space. After two minutes of acclimation time, one sheet was removed to reveal a 60 × 60 mm door at the bottom of the other sheet, large enough for any of the fish to comfortably swim through. The movement of fish was tracked for 5 min after the door was opened. For video analysis, the “revealed” part of the arena was divided into a 3 × 3 grid, and the acclimation zone was defined as a tenth zone. Exploratory behavior was quantified by calculating Simpson's evenness from the proportion of time spent in each of the 10 zones, with higher evenness interpreted as a more thorough exploration of the space. This measure gives the lowest score to fish that never enter the revealed space, but does not have the same statistical issues related to censored data as measures based on latency to enter.

Repeatability of activity (mean velocity in m/s) and exploratory behavior (evenness of zone visitation) was calculated using the “rptGaussian” function in the “rptR” R package (Bell, Hankison, & Laskowski, 2009; Stoffel, Nakagawa, & Schielzeth, 2017). The mean of the two trials was calculated for each behavior for each individual, the correlation between behaviors was tested, and the individual means were used in subsequent tests for carryover effects. After the behavioral assays, fish were euthanized with an overdose of AQUI‐S and preserved in 90% ethanol for dissection.

We measured “behavior” fish for TL and then dissected them to measure parasite load, which can be correlated with behavioral traits in bullies (Hammond‐Tooke, Nakagawa, & Poulin, 2012). All tissues excluding the head were inspected thoroughly, and the total number of three trematodes (cysts of *Apatemon* sp., *Telogaster opisthorchis*, and *Stegodexamene anguillae*) and one nematode (*Eustrongylides* sp.) was counted.

### 2.4 Diet and parasitism

Both gut content and stable isotopes were used to infer the trophic niche of the “diet” subset of fish from Lake Waipori. To assess short‐term diet, the digestive tract was removed and prey items in the stomach and intestine were identified under a dissecting microscope to broad taxonomic categories: chironomid larvae, amphipods, isopods, mysid shrimps and gastropods. To assess diet of adult fish over a longer time frame (likely 1–3 months), we used carbon and nitrogen stable isotope analysis of fish and prey items. A small piece of posterior dorsal muscle was removed from each fish. Invertebrates collected from the lake were sorted into the same taxonomic groups as the diet data. Small mud snails were acidified to remove inorganic carbon from the shell, following Jaschinski, Hansen, and Sommer (2008): we added 0.2 µl of 10% HCl, dried the sample one hour at 60°C, added another 0.2 µl of 10% HCl, and dried again the sample 12 hr at 60°C. Each sample was dried for 48 hr at 60°C and homogenized with a mortar and pestle, then a ~0.8 mg sample was weighed into a tin capsule. Samples were analyzed for δ^13^C and δ^15^N on a Europa Hydra stable isotope mass spectrometer (Europa 126 Scientific) interfaced to a Carlo Erba elemental analyser (NC2500; Carlo Erba) at the Iso‐trace laboratory (Department of Chemistry, University of Otago). Isotope values are expressed in the standard delta notation relative to the international standards Pee Dee Belemnite limestone carbon and atmospheric nitrogen. Precision based on internal standards was ±0.3 ‰ for δ^15^N and ±0.2 ‰ for δ^13^C, and analysis of replicate samples showed high repeatability (*R* = .97 for δ^15^N and .99 for δ^13^C). We applied a standard arithmetic formula to normalize δ^13^C to account for the effect of lipids in depleting ^13^C relative to the diets of aquatic organisms (Post et al., 2007).

### 2.5 Otolith aging and microchemistry

Sagittal otoliths were gently extracted, soft tissues were removed when present, and otoliths were rinsed using purified water. Otoliths were mounted sulcus side up on glass slides using a small drop of Crystalbond 509 (SPI supplies) melted at approximatively 60°C. Left and right otoliths were randomly assigned to either microchemistry or aging.

For aging, the sulcus side of the otolith was sequentially polished using 2,000 and 1,000 grit sandpaper then 30 µm and 3 µm lapping film. Otoliths from one‐year‐old tank‐reared bullies (offspring of parents from the same population) were used to confirm landmark growth increments. A metamorphosis check was visually assigned based on the presence of a characteristic fine dark band around the core (Shen & Tzeng, 2002), and the observation of some accessory primordium (Daverat, Martin, Fablet, & Pécheyran, 2011). This check was usually followed by an opaque area in wild‐caught fish. Dark and opaque annuli were interpreted as winter growth checks (Jellyman, Sagar, Glova, & Sykes, 2000). Distances between the core, metamorphosis check, annual increments, and the edge were measured using AmScope software (AmScope), along an axis running between the core and the fastest‐growing posterior dorsal edge. Distances were measured by only one observer (GS) and were blind with regard to microchemistry data to minimize any observer effects or bias. We used otolith growth to infer fish growth (Ashworth, Hall, Hesp, Coulson, & Potter, 2016), which has been validated in the closely related *G. gobioides* (Jellyman et al., 2000). Based on previous studies indicating a summer spawning period for *G. cotidianus* on the South Island (McDowall, 1990b) and the observation of many ripe male and female gonads, we were confident that we had sampled fish approximately one year of age.

For microchemistry, a depth profile of element concentrations (Li^7^, Mg^24^, Al^27^, P^31^, Mn^55^, Cu^63^, Sr^88^, Ba^138^) was measured using an ArF excimer ultraviolet (UV) laser system (193‐nm wavelength; Resonetics LPX120i ArF, Resonetics LLC) coupled to a quadrupole ICP‐MS (Agilent 7500s Series, Agilent Technologies) at the Centre for Trace Element Analysis at the University of Otago. The laser operated at a repetition rate of 10 Hz, with a spot size of 90 µm and energy of 3 mJ. The depth profile was chosen to go through the core and drilling was aborted when the laser had passed through the core or the entire otolith (characterized by a sudden drop in Ca and increase of metal elements). NIST standards 610, 612, and MACS3 were run every 5–6 otoliths. Data were processed using Igor Pro software (Wavemetrics, Lake Oswego, OR, USA): standards were controlled, and otolith data were calibrated according to standard levels. Data were smoothed by a factor of 5 (i.e., time step standardized to 1 s). The core position was visually assigned based on the symmetry of Ba^138^ and Sr^88^ profiles, sometimes by the presence of a characteristic spike of Mn^55^ (Ruttenberg et al., 2005), and by the empirical observation of a large spike of P^31^ and Mg^24^ following the core, interpreted as the protein‐rich metamorphosis check (Figure 1c; Shen & Tzeng, 2002).

As the otolith growths, it traps minor elements, and Ba and Sr are believed to randomly substitute for Ca in the otolith aragonite matrix (Doubleday, Harris, Izzo, & Gillanders, 2014). Otolith ratios of Ba/Ca and Sr/Ca have been shown to be a powerful tool to reconstruct past environments experienced by fish (Macdonald & Crook, 2010). High Sr and low Ba ratios are indicative of saline environments, while low Sr and high Ba typically indicate freshwater habitats (Zimmerman, 2005). Relationships between environmental Sr/Ca and otolith Sr/Ca may be affected by extrinsic factors such as temperature or salinity, and by intrinsic factors such as life stage or metabolism (Macdonald & Crook, 2010; Zimmerman, 2005). However, most laboratory experiments support the idea that otolith Sr/Ca is mainly determined by Sr/Ca water concentration (Hicks, Closs, & Swearer, 2010; Walsh & Gillanders, 2018), and thus reflective of salinity environment. In addition, the partition coefficient of Sr, and thus the relationship between environmental and otolith Sr/Ca, is generally robust to slight variation in salinity (Hicks, 2012; Zimmerman, 2005). Larval Sr^88^ was measured as the mean value across 5 s of drilling on each side of the core. Adult Sr^88^ was measured as the mean value between 10 and 30 s of drilling, to avoid contamination from the surface of the otolith and to average over a substantive and consistent time period, estimated at between one and two months (Figure 1c).

To investigate the relationship between environment and otolith Sr/Ca, we pooled fish into five groups depending on their capture site: upstream (*N* = 10), downstream (*N* = 10), and three locations within the lake: North (*N* = 17), South (*N* = 26), and East (*N* = 15) (Figure 1a–d). We compared adult Sr/Ca between groups using an ANOVA. To allow comparison with average salinity measured over a longer period, we matched our sites (within 1 km) to locations from an earlier study, assuming that the salinities in the system have not changed significantly over two decades (Schallenberg & Burns, 2003). Except for site EL for which we did not have data, our sites UL, NL, SL, and DL matched sites with mean annual salinities of 0, 0.5, 0.9, and 2.1 ppt (Schallenberg & Burns, 2003).

### 2.6 Data analysis

To investigate carryover effects on growth across life stages, we used path analysis (i.e., structural equation modeling; Figure 3), which allows comparison of direct and indirect effects between sets of multiple variables. To remove annual environmental variability, only the one‐year‐old cohort of fish was analyzed. As otolith microchemistry did not reveal discrete cohorts corresponding to marine and freshwater‐reared fish (see Results), we used both larval and adult otolith Sr/Ca as continuous measures of environment, expected to correspond (albeit imperfectly) to salinity. We included five variables in our models: larval and adult Sr/Ca, and growth of the three life stages (larval, juvenile, and adult). We tested three different hypothesis: (a) growth can carry over to affect growth later in development (Figure 3a), (b) environmental salinity (i.e., otolith Sr/Ca) can carry over to impact growth at a later stage (Figure 3b), both past growth and environmental salinity carryover to impact later growth (Figure 3c). Models were implemented using the R package piecewiseSEM (Lefcheck, 2016). Otolith Sr/Ca was included in model 1 (i.e., the model was nested), to allow comparison with the two other models. Model fits were evaluated using Shipley's test of d‐separation test using Fisher's C statistic (Shipley, 2013). Model fits were compared using the Akaike Information Criteria corrected for small sample size (AIC_c_) as implemented in the R package piecewiseSEM (Lefcheck, 2016). ∆AIC_c_ was calculated for each model as the difference with the lowest AICc that we found. Models with ∆AIC_c_ > 3 were considered to have substantially less support (Burnham, Anderson, & Burnham, 2002).

To analyze diet composition data obtained from gut contents, we performed a redundancy analysis (RDA; van den Wollenberg, 1977) using the R package vegan (Oksanen, 2018). We used all “diet” fish with a minimum of three prey items, and as constraining variables, we used larval Sr/Ca, adult Sr/Ca, their interaction, and fish TL (Figure 4c). RDA allows regression of multiple response variables on parameters and is a powerful tool for investigating community composition as a response variable (Ramette, 2007). By treating the stomach contents of individuals as “community” data, RDA allows us to analyze multivariate diet responses to combinations of linear predictors. We used a permutation test to measure the marginal effect of each predictor and to calculate statistical significance.

To investigate carryover effects of natal habitat on adult traits, we used linear models with larval Sr/Ca, adult Sr/Ca, their interaction, and fish TL as predictors. As response variables, we used δ^15^N, normalized δ^13^C, activity (mean velocity), exploration (evenness of zone visitation), and total load of each of four parasites: three trematodes (cysts of *Apatemon* sp., *Telogaster opisthorchis*, *Stegodexamene anguillae*) and one nematode (*Eustrongylides* sp.). Normality of residuals and homogeneity of variances were checked for each analysis. All data analysis was carried out using R (R Core Team, 2017).

To investigate correlations between *Apatemon* sp. load and behavior, as well as between larval and adult Sr/Ca, we performed a Kendall correlation test.

## 3 RESULTS

### 3.1 Water chemistry analysis

Water analysis across the system revealed that, as expected, Sr/Ca was highly correlated with log‐transformed salinity (Figure 1b, *R* ^2^ = .82, *p* < .00001, Sr/Ca = 0.90*log(salinity) + 6.83). In contrast, local variation in Ba/Ca was less predictable and may have been influenced by heterogeneous inflows of Ba‐enriched water from the upper Waipori and Taieri Rivers (data not shown). Therefore, we excluded Ba/Ca and used only Sr/Ca as an indicator of salinity environment in the following analysis.

### 3.2 Past environmental history of fish

Adult Sr/Ca varied significantly among fish capture locations (Figure 1d, ANOVA, *p* = .00018). Adult Sr/Ca was positively correlated with mean salinity from 1997 to 1998 across the four sites for which we had matching water chemistry data (log‐linear relationship, *R* ^2^ = .75, *p* = .037). Globally, adult Sr/Ca increased from upstream to downstream in the system (Figure 1d). Fish caught upstream of the lake had all low adult Sr/Ca (<0.85 mmol/L), consistent with the absence of saline intrusion. In contrast, the highest adult Sr/Ca values were found downstream of the lake, where salinity is expected to be the highest. Yet, some fish from downstream and the eastern part of the lake, displayed a very low adult Sr/Ca (<0.5 mmol/L), potentially due to the proximity of several freshwater tributaries from which fish could had recently migrated. Based on these lines of evidence, we cautiously interpret otolith Sr/Ca as an indicator of environmental salinity in the system in the following analyses.

**Figure 2:**
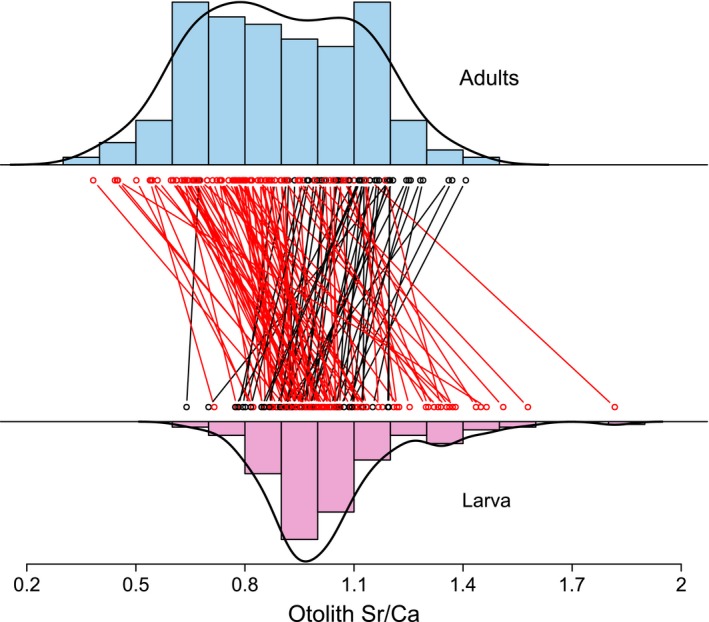
Histogram of adult (top, blue) and larval (bottom, pink) of otolith Sr/Ca. Lines connect points representing the same individual. Increases and decreases in Sr/Ca from larval to adult stages are represented by black and red lines, respectively

We did not identify any fish with clearly amphidromous life histories. Only one fish showed a high larval Sr/Ca signature (>1.8; Figure 2), but this was not associated with decreased Ba as is characteristic of sea residence. There was an overall trend for fish to show decreased Sr/Ca from larval to adult stages (paired *t* test, *p* < .00001), consistent with an amphidromous‐like lifestyle involving an larval downstream migration (Figure 2). While adult and larval Sr/Ca were positively correlated (Kendall correlation test, *p *= <.001), the correlation between them was low (Kendall correlation coefficient = 0.24), and shifts consistent with both upstream and downstream ontogenetic movements were observed (Figure 2). Thus, our results suggest a continuum of life histories with larvae rearing between the lake and the lower Taieri estuary.

### 3.3 Fish growth

Of the aged fish (*N* = 102), most were 1 + years old (*N* = 89), while relatively few were young‐of‐year (age 0+, *N* = 2) or age 2+ (*N* = 11). To avoid effects of interannual environmental variation, the relationship between otolith microchemistry and growth was examined only for one‐year‐old fish from Lake Waipori.

**Figure 3:**
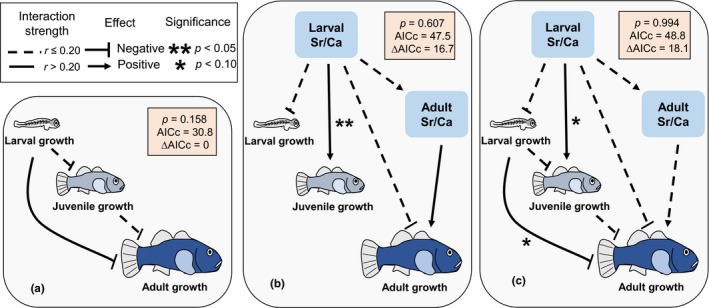
Path analysis of traits across life stages in common bully from Lake Waipori. Three different models were considered: growth is affected by past growth (a), growth is affected by past environmental history (b), and growth is affected by both past growth and environmental history (c). Each line represents an hypothetical interaction between variables that was considered in the model. We reported for each model the *p*‐value of the fit, the Akaike Information Criteria corrected for small sample size (AIC_c_), and its difference with model 1 (i.e., ∆AIC_c_)

Path analysis which included environmental salinity had substantially less support (∆AIC_c_ > 10, Figure 3), suggesting that salinity has a minor impact on fish growth.

### 3.4 Fish diet

**Figure 4:**
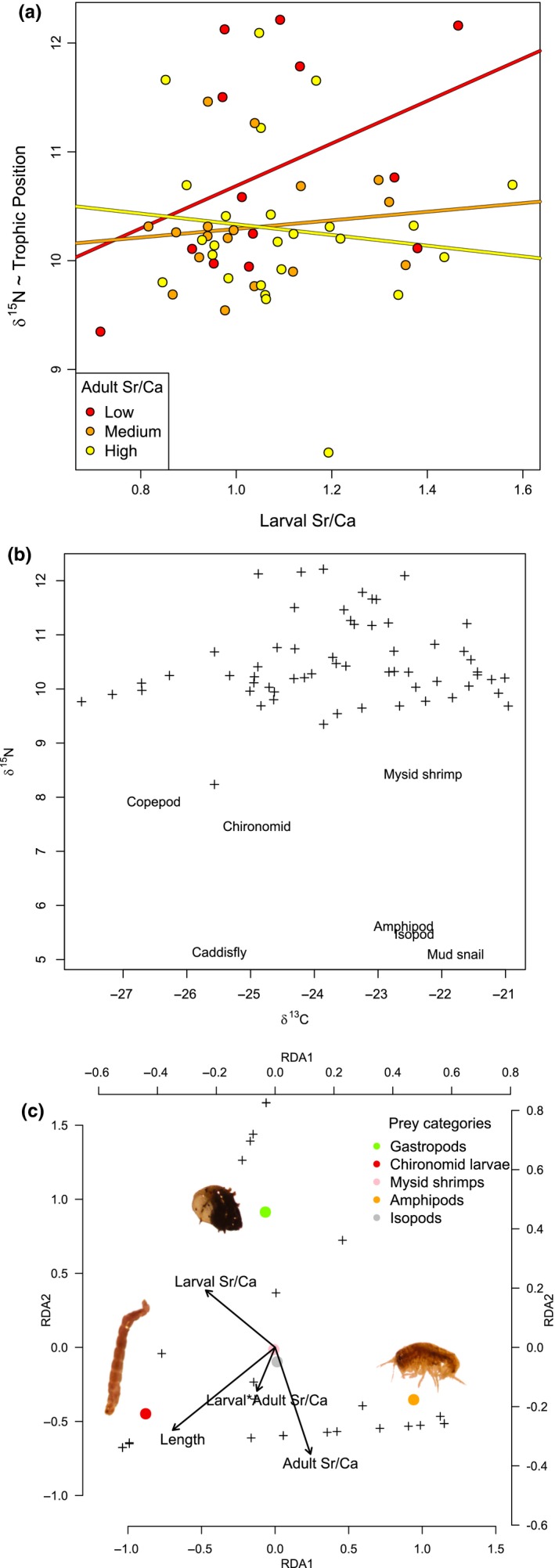
Diet analysis of common bully from Lake Waipori. Individuals fish are represented by black crosses. (a) Interactive effect of larval otolith Sr/Ca and adult otolith Sr/Ca on δ^15^N in adult bullies. For the purpose of illustration of the interaction, points are colored, and regression lines are shown for three equal‐sized groups with low, medium and high adult Sr/Ca values (b) Carbon/nitrogen stable isotope analysis of common bully and prey types in Lake Waipori. (c) RDA analysis on gut content of common bully, constrained by fish size, larval and adult otolith Sr/Ca, and their interaction. Bottom and left axis: RDA values of fish and RDA axes; top and right axis: RDA values of prey. Colored dots are prey categories (see legend).

Fish showed a wide range of δ^15^N and δ^13^C, consistent with the range of prey isotope values and with trophic enrichment primarily in δ^15^N (Figure 4b; Gu, Schelske, & Hoyer, 1996). Lipid‐normalized δ^13^C was not significantly related to any predictor, though there was a non‐significant negative relationship between fish length and δ^13^C. δ^15^N was significantly predicted by larval Sr/Ca and its interaction with adult Sr/Ca (Table 1). This result indicates that trophic position is influenced by the interplay between an individual's present and past environment and is highest in fish with high larval Sr/Ca and low adult Sr/Ca (Figure 4a). Of the prey items sampled, higher values of δ^13^C separated amphipods and gastropods from chironomid larvae, while the highest δ^15^N values came from copepods, shrimp, and chironomid larvae.

**Table 1:** Effects of larval and adult environment, and length on adult traits of *G. cotidianus*

Variable	Regression coefficient (*p*‐value)				Sample size
	Larval Sr/Ca	Adult Sr/Ca	Larval Sr/Ca × adult Sr/Ca	Length	
δ^13^C	+1.6 (0.74)	+4.8 (0.41)	−1.7 (0.76)	−0.06 (0.10)	50
δ^15^N	+5.5 (0.029*)	+5.16 (0.086)	−5.71 (0.042 *)	0.00 (0.70)	50
*Apatemon* sp	+1,182 (0.0019**)	+976 (0.0099**)	−1,082 (0.0046**)	+7.1 (0.012*)	77
*Telogaster opisthorchis*	+6.6 (0.45)	+7.7 (0.41)	−7.6 (0.42)	−0.01 (0.76)	61
*Stedodexamene anguillae*	+7.9 (0.36)	+7.2 (0.42)	−6.5 (0.48)	0.00 (0.90)	61
Nematode	−1.1 (0.23)	−0.70 (0.66)	+0.76 (0.63)	+0.01 (0.31)	77
Evenness	−0.06 (0.91)	−0.09 (0.87)	+0.05 (0.931)	0.00 (0.40)	60
Velocity	−1.5 (0.49)	−0.66 (0.76)	+0.99 (0.65)	−0.2 (0.15)	58
Gut contents (RDA)	0.328	0.188	0.758	0.034*	52

Note

Linear models were used to test the significance of each predictor on each adult trait, except for gut content composition which was analyzed using redundancy analysis followed by a permutation‐based ANOVA. Statistical significance is reported.

* *p* < .05.

** *p* < .01.

Of the fish analyzed for gut contents (*N* = 52), 47 had nonempty guts which contained an average of 13 diet items. Bully diets were mostly composed of chironomid larvae (37.2%), amphipods (34.9%) and gastropods (26.2%), with few isopods (1.6%) and mysid shrimps (0.1%; Figure 4c). The only significant predictor in the RDA was TL (*p* = .034), which was most associated with feeding on chironomid larvae. Though larval and adult Sr/Ca were not significant predictors, amphipods were most associated with high adult Sr/Ca, while gastropods were most associated with high larval Sr/Ca.

### 3.5 Parasite infection

**Figure 5:**
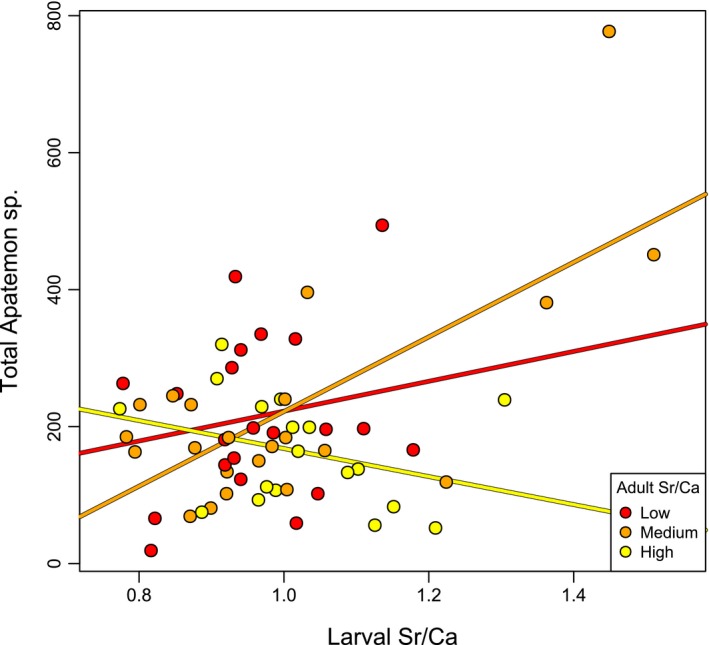
Interactive effect of larval otolith Sr/Ca and adult otolith Sr/Ca on *Apatemon* sp. load in adult bullies. For the purpose of illustration of the interaction, points are colored, and regression lines are shown for three equal‐sized groups with low, medium, and high adult Sr/Ca values

All the fish dissected (*N* = 81) were infected by *Apatemon* sp., with loads ranging from 7 to 777 cysts per individual (mean = 178). *Apatemon* sp. was by far the most abundant parasite, as mean parasite loads were only 2.3, 2.2, and 0.1 per fish for *Telogaster opisthorchis*, *Stegodexamene anguillae*, and nematodes, respectively. *Apatemon* sp. load was significantly explained by larval Sr/Ca and its interaction with adult Sr/Ca (Table 1) and was highest in fish with higher larval Sr/Ca and lower adult Sr/Ca (Figure 5). We did not find any influence of larval or adult Sr/Ca on rates of infection by the other three parasites (Table 1).

### 3.6 Behavioral assays

Both traits showed modest but statistically significant individual‐level repeatability (activity: *R* = .269, 95% CI = 0.029–0.487, *p* = .018; exploratory behavior: *R* = .242, 95% CI = 0–0.462, *p* = .031). There was a significant positive correlation between activity and exploration across individuals (*r* = .385, *p* = .002). We did not detect any influence of larval Sr/Ca, adult Sr/Ca, or total length on either activity or exploratory behavior (all *p* > .1; Table 1). In addition, *Apatemon* sp. load was not correlated with exploratory (*p* = .85, *r* = .17) and activity behavior (*p* = .33, *r* = −.09).

## 4 DISCUSSION

Our study suggests that environment early in the life of a facultatively amphidromous fish influences some but not all forms of between‐individual variation in the adult population. The common bully has a complex life cycle typical of amphidromous fish, with larvae using pelagic habitats followed by a transition to a benthic lifestyle after metamorphosis, usually accompanied by some form of migration. Traditionally, metamorphosis is viewed as a strategy to break down ontogenetic constraints in order to optimize fitness at each developmental stage (Campero, De Block, Ollevier, & Stoks, 2008; Phillips, 1998). However, our study supports the more recent idea that metamorphosis is a porous boundary between developmental stages and that larval experience can influence adult phenotypes or fitness (Crean, Monro, & Marshall, 2011; Pechenik, 2006). The underlying mechanism of carryover effects remains generally unknown, likely because it is tied to the complex series of events associated with development and metamorphosis. Several studies have revealed the role of genetic architecture which binds traits across life stages (Aguirre, Blows, & Marshall, 2014), while evidence for environment‐specific covariations of genes across life stages (Gutteling et al., 2007) suggests that carryover effects are themselves environment‐specific. Regardless of the mechanisms behind carryover effects, their existence demonstrates how life stages are correlated and sheds light on how individual variation can manifest across life history.

Consistent with previous studies in the Waipori–Taieri system (Closs et al., 2003; Hicks et al., 2017), we observed Ba signatures that remained high in otolith cores and low concentrations of Sr compared to diadromous common bully (Hicks, 2012), and we could not conclude that any fish were purely diadromous. We instead found continuous variation in Sr/Ca of both larval and adult otoliths, which we cautiously interpret as variation along a salinity gradient from estuarine to pure freshwater. We observed a 3.6‐fold variation of larval otolith Sr/Ca between 0.5 and 1.8. Comparing with adult otolith edge values (Figure 1d), the lowest values may correspond to freshwater with negligent salinity (0.0 ppt). Though we do not have experimental experiments on common bullies to predict the variation with salinity of the Sr partition coefficient in otoliths, it is not expected to vary widely (Hicks et al., 2010; Taddese, Reid, & Closs, 2019; Zimmerman, 2005). Based on the relationship between environmental Sr/Ca and salinity, and empirical data from edge otolith values, we estimate that larva may have experienced salinities between 0 and 10–15 ppt. Such variation of salinity will affect numerous aspects of water quality as well as the availability or prey such as zooplankton (Schallenberg & Burns, 2003). We acknowledge that some variation in Sr/Ca may come from temporal or microhabitat variation in salinity rather than position on the broad salinity gradient and that other aspects of larval environment, such as water depth and flow, are not captured by our proxy for larval environment.

### 4.1 Carryover effects of early life environment

We did not find a clear immediate or latent effect of salinity on growth. This shows that larval growth can be achieved in a wide range of salinity, supporting the idea that the resource requirements of some amphidromous fishes can be fulfilled in freshwater and brackish environments as well if plankton resources are available (Augspurger, Warburton, & Closs, 2017). In temperate latitudes, according to the “food availability hypothesis” fish migrate to saline habitats to benefit from a more productive ecosystem (Gross et al., 1988) and feeding opportunities often explain migration in diadromous fishes (Chapman et al., 2012). As illustrated by recent findings in white perch (Gallagher et al., 2018), the benefit of migration can be expressed latter in time through carryover effects. As we focused on a single year, we might also have failed to detect significant effects because of annual variability. For instance, there is year‐to‐year variation in the growth advantage of resident versus migratory black bream (Gillanders et al., 2015).

We found that diet was influenced by early life environment. Specifically, fish trophic position, as inferred using δ^15^N, was affected by the interaction between larval and adult Sr/Ca. One explanation is that fish that migrated upstream were more likely to eat prey such as copepods, shrimp, and chironomid larvae, although only the latter was common in gut contents at the time of sampling. δ^15^N values in consumers can be influenced by factors other than diet, such as nutrient stress (Bowes, Lafferty, & Thorp, 2014; McCutchan, Lewis, Kendall, & McGrath, 2003), so we cannot be certain as to the mechanism behind the patterns of variation in δ^15^N. δ^13^C was not related to any measure of salinity environment, though the negative relationship with TL indicates some partitioning of diet among size classes. Gut content data did not yield significant relationships, but suggested some variation in diet with adult environment (i.e., gastropods are replaced by amphipods with increased salinity), as well as a weak interactive effect of larval and adult environment (fish with distinct larval and adult environments tended to feed more on chironomid larvae).

We found that larval salinity environment can carry over to affect fish vulnerability to a trematode, *Apatemon* sp. Globally, infection rates are higher in upstream areas with lower salinities. This result supports findings in another bully species, *Gobiomorphus breviceps*, in which *Apatemon* sp. infection does not linearly decrease upstream but is more locally influenced. Its intermediate host, the mud snail *Potamopyrgus antipodarum* (Poulin, Hammond‐Tooke, & Nakagawa, 2012), is expected to be more abundant in less saline water despite a broad salinity tolerance (Gerard, Blanc, & Costil, 2013). This is consistent with our diet data, though we do not have direct snail abundance data. Thus, salinity may indirectly influence *Apatemon* sp. infection through mud snail habitat preference. However, fish that experienced more saline habitats as larvae had higher infection rates, suggesting that contact with the parasite in early life may be necessary to develop defence against it, resulting in a “mismatch effect” (Hoverman & Relyea, 2009). This contrasts with findings that amphidromous galaxiid fish in New Zealand are advantaged over freshwater‐resident congeners by reduced vulnerability to a different trematode parasite (Poulin, Closs, et al., 2012). Our results suggest that adult parasite infection rates are more than the accumulation of parasites throughout life. For instance, the highest infection rates were found in fish coming from high salinities, in which infection transmission is expected to be lower. Therefore, *Apatemon* sp. load is not fully representative of habitat use throughout life history, which could complicate the use of parasite load to assess long‐term habitat use (McCairns & Fox, 2004; Wilson, Coleman, Clark, & Biederman, 1993). Parasite infection can profoundly affect fish fitness through energy cost, as parasite mass can reach 20% of total fish biomass. Yet, the relationship between Apatemon sp. abundance and body condition is unclear as it suggested that intermediate parasite load is beneficial for common bully body condition (Maceda‐Veiga, Green, Poulin, & Lagrue, 2016). Host manipulation may also play a role; it is suggested that *Apatemon* sp. infection increases aggressiveness and therefore vulnerability to avian predators (Hammond‐Tooke et al., 2012).

Previous studies have shown that behavioral traits such as boldness are associated with the propensity to migrate, for instance in roach (Chapman, Hulthén, et al., 2011). Interestingly, we did not find any correlation between measured behavioral traits (activity and exploration) and environmental history. Given that the initial migration downstream is carried out by weak‐swimming larvae rather than adults in this species, it may be that adult personality is decoupled from the extent of migration early in life. More surprising is that neither the larval habitat nor the inferred extent of upstream migration showed any relationship with behavior. It may be that the traits measured do not capture the tendency of individuals to migrate between habitats or that individual histories are more dictated by chance movement (e.g., during floods and spring tides) than by any repeatable behavioral tendencies.

### 4.2 Life‐history flexibility

The weight of evidence suggests that there is no clear advantage of any single life history in this system. Fish that move upstream into lower salinity water seem to be affected by increased rates of parasitism via a mismatch effect. In addition, larval experience seems contribute to variation in adult resource use, as larval environment impacts adult diet including trophic position. Therefore, it is unclear what drives variability of migratory patterns in the system. It is more and more evident that migration is an heterogeneous phenomenon (Chapman et al., 2012), but while it has long been proposed that migratory strategies are adaptations to maximize individual fitness (Lundberg, 1988), no straightforward model answers this question (Chapman, Brönmark, et al., 2011). Some aspects of life history have not been included in our study, such as survival, which is often involved in migratory trade‐off strategies (Dodson, Aubin‐Horth, Thériault, & Páez, 2013; Thériault, Dunlop, Dieckmann, Bernatchez, & Dodson, 2008). If particular larval habitats lead to substantially reduced survival rates, these effects could dwarf the relatively subtle effects we found on parasitism and diet. Also, we only sampled a single year, whereas interannual variation may result in an equilibrium migratory strategy that maximizes expected fitness across years. For instance, the fitness outcome of carryover effects induced by migration in white perch varies between years (Gallagher et al., 2018), which could promote coexistence of strategies (Gillanders et al., 2015). In addition, our study focuses on a single river system while the benefits of different life histories are likely to vary with environmental context. A recent study shows that the boundary between freshwater and seawater habitats affects growth rates of migrating juveniles of another New Zealand amphidromous fish, *Galaxias maculatus* (Kaemingk, Swearer, Bury, & Shima, 2019). This suggests that carryover effects are themselves variable among river systems, which adds another source of individual variation at large spatial scales.

The limited carryover effect of larval habitat on adult habitat choice (Figure 2) is unexpected and suggests that patterns of individual migration are complex. The highly flexible larval environment and frequent ontogenetic habitat shifts in this species might maintain connectivity across salinity environments, with potential implications for ecosystem functioning (Sheaves, 2009). The absence of full amphidromy (i.e., migration to the ocean) that we found is surprising given the direct access to the sea and the presence of marine‐reared individuals in this and similar systems in previous years (Closs, Hicks, & Jellyman, 2013). Forecast impacts of climate change include altered patterns of salinity in coastal lakes and estuaries (Schallenberg & Burns, 2003), and our study highlights the cascade of consequences that could result from carryover effects of altered larval environments.

## 5 CONCLUSION

Our study shows that variation of one environmental factor in early life‐history generates individual variation in natural populations. This in turn may allow population niche expansion, increased between‐individual variation, and population resilience through the portfolio effect (Bolnick et al., 2003; Dall et al., 2012). An appreciation of carryover effects may therefore be crucial to our understanding of how individual variation influences population dynamics, and how disruption of migration due to flow regulation and habitat modification could have cascading consequences due to the loss of carried‐over traits.

## CONFLICT OF INTEREST

None declared.

## AUTHOR CONTRIBUTIONS

TI conceived the study; GS and TI designed the study; GS collected and analyzed the data; GS led the writing of the manuscript with contributions from TI. Both authors approved submission of the manuscript.

## Data Availability

Data are available from the Dryad Digital Repository: https://doi.org/10.5061/dryad.852h5m8.
